# Decrease in abundance of bacteria of the genus *Bifidobacterium* in gut microbiota may be related to pre-eclampsia progression in women from East China

**DOI:** 10.29219/fnr.v65.5781

**Published:** 2021-06-28

**Authors:** Tingting Miao, Yun Yu, Jin Sun, Aiguo Ma, Jinran Yu, Mengjun Cui, Liping Yang, Huiyan Wang

**Affiliations:** 1 Department of Education, Changzhou Maternity and Child Health Care Hospital Affiliated to Nanjing Medical University, Changzhou, China; 2 Institute of Nutrition and Health, Qingdao University, Qingdao, China; 3 Department of Clinical Laboratory, Changzhou Maternity and Child Health Care Hospital Affiliated to Nanjing Medical University, Changzhou, China; 4 School of Public Health, Qingdao University, Qingdao, China; 5 School of Food Science and Technology, Jiangnan University, Wuxi, China; 6 Department of Obstetrics and Gynecology, Changzhou Maternity and Child Health Care Hospital Affiliated to Nanjing Medical University, Changzhou, China

**Keywords:** *gut microbiota*, Blautia, Ruminococcus, Bifidobacterium, *pre-eclampsia*

## Abstract

**Background:**

Pre-eclampsia (PE) can result in severe damage to maternal and fetal health. It has been reported that gut microbiota (GM) had important roles in regulating the metabolic and inflammatory responses of the mother. However, investigations on GM in PE are rare.

**Objective:**

The objective of the present study was to investigate the changes of GM in PE and how to alter the GM composition in PE by dietary or dietary supplements.

**Design:**

We analyzed the composition changes in GM as well as the relationship between bacteria of different genera and clinical indices by amplifying the V4 region of the 16S ribosomal RNA gene in 12 PE patients and eight healthy pregnant women in East China.

**Results:**

In the PE group, the Observed Species Index was lower than that in the control group, indicating that the α-diversity of the microbiome in the PE group decreased. At phylum, family, and genus levels, the relative abundance of different bacteria in PE patients displayed substantial differences to those from healthy women. We noted a decreased abundance of bacteria of the phylum Actinobacteria (*P* = 0.042), decreased abundance of bacteria of the family Bifidobacteriaceae (*P* = 0.039), increased abundance of bacteria of the genus *Blautia* (*P* = 0.026) and *Ruminococcus* (*P* = 0.048), and decreased abundance of bacteria of the genus *Bifidobacterium* (*P* = 0.038)*.* Among three enriched genera, bacteria of the genus *Bifidobacterium* showed a negative correlation with the systolic blood pressure (SBP), diastolic blood pressure (DBP), and dyslipidemia, which involved glucose metabolism, lipid metabolism, and the oxidative-phosphorylation pathway. The increased abundance of bacteria of the genera *Blautia* and *Ruminococcus* was positively correlated with obesity and dyslipidemia, which involved lipid metabolism, glycosyltransferases, biotin metabolism, and the oxidative-phosphorylation pathways. Moreover, women in the PE group ate more than women in the control group, so fetuses were more prone to overnutrition in the PE group.

**Conclusion:**

There is a potential for GM dysbiosis in PE patients, and they could be prone to suffer from metabolic syndrome. We speculate that alterations in the abundance of bacteria of certain genera (e.g. increased abundance of *Blautia* and *Ruminococcus*, and decreased abundance of *Bifidobacterium*) were associated with PE development to some degree. Our data could help to monitor the health of pregnant women and may be helpful for preventing and assisting treatment of PE by increasing dietary fiber or probiotics supplement.

## Popular scientific summary

- Pre-eclampsia is a common hypertensive disorder during pregnancy which may be related to the alternaions of gut microbiota, however investigations on gut microbiota in pre-eclampsia are rare.
- Decreased in abundance of bacteria of the genus *Bifidobacterium* showed a negative correlation with the systolic blood pressure, diastolic blood pressure, and dyslipidemia.
- Our data could help to monitor the health of pregnant women and may be helpful for preventing and assisting treatment of pre-eclampsia by increasing dietary fiber or probiotics supplement.

Pre-eclampsia (PE) is a common hypertensive disorder during pregnancy. It affects an estimated 3–5% of pregnancies worldwide and contributes substantially to the morbidity and mortality of the mother and fetus (1). One characteristic of PE is the development of concurrent hypertension and proteinuria or end-organ damage in middle- or late-stage pregnancy (2). Various angiogenic, genetic, structural, and metabolic pathways have been considered to be related to PE development, including remodeling of spiral arteries, placental oxygenation, and immune tolerance (2, 3). Endothelial dysfunction as well as an imbalance of angiogenic and antiangiogenic factors during pregnancy is associated with PE (2, 4).

Gut microbiota (GM) consist of a complex and diverse community of microorganisms (5). In health, human GM interact and balance in abundance with each other to form a stable ecosystem. The latter plays a unique part in metabolism, immunity, and nutrition absorption (6). There are ~1,014 known bacteria in the intestine, including >2,000 species and 12 phyla (7). GM can produce diverse compounds that can regulate the activities of distal organs. These actions could facilitate insulin resistance by inducing chronic inflammation, which has an important role in disease development (6). The various factors that affect intestinal microecology, such as consumption of a high-fat diet, obesity, and an excessive increase in the gestation period, are independent risk factors for cardiovascular disease and metabolic disease. Otherwise, these factors can induce metabolic abnormalities, excessive oxidative stress, and immunotolerance imbalance by changing intestinal microecology and alterations in abundance of specific bacteria, which can result in the occurrence and development of PE.

However, the relationship between the diversity of intestinal microflora and PE in women from East China is not known (although some results are available for women in South China [8]). Here, we analyzed GM differences between women with PE and healthy pregnant women (control group). We also assessed the relationship between the abundance of different GM and clinical indices. In this way, we offered new concepts on the prediction of PE and potential interventions against PE.

## Materials and methods

### Ethical approval of the study protocol

The study protocol was approved by the Internal Ethics Committee of the Institute of Chinese Medical Sciences, and Ethics Committee of Changzhou Maternity and Child Health Care Hospital (QNRC2016302) in Changzhou, China. The study was carried out in accordance with the Declaration of Helsinki 1964 and its later amendments. Patients provided written informed consent for collection of their stool samples for experimentation.

### Patients

Patients were treated in the Department of Obstetrics and Gynecology in Changzhou Maternity and Child Health Care Hospital (which is affiliated to Nanjing Medical University) from October 2017 to April 2018. According to the current guidelines from American College of Obstetricians and Gynecologists (ACOG) (2019), the diagnostic criteria for PE were as follows: 1) systolic blood pressure (SBP) ≥ 140 mmHg and/or diastolic blood pressure (DBP) ≥ 90 mmHg on two occasions at least 4 h apart after 20 weeks of gestation in a woman with a previously normal blood pressure; 2) proteinuria > 300 mg/24, or protein/creatinine ratio ≥ 0.3 mg/dL, or dipstick reading of 2+ (used only if other quantitative methods not available); 3) in the absence of proteinuria, new-onset hypertension with the new onset of any of the following: a) thrombocytopenia: platelet count < 100,000×109/L; b) renal insufficiency: serum creatinine concentrations> 1.1 mg/dL or a doubling of the upper limit in the absence of other renal disease; c) impaired liver function: elevated blood concentrations of liver transaminases to twice normal concentration; d) pulmonary edema; e) new-onset headache unresponsive to medication and not accounted for by alternative diagnoses or visual symptoms (2, 9). The age of pregnant women, gestational weeks when the fecal samples were collected, and gravidity and parity were matched in the two groups (10). None of the women in either group had: 1) gestational diabetes mellitus, hyperlipidemia, or other complications of pregnancy; 2) an infection or used antibiotics in the month before stool collection.

### Sample collection and microbiome sequencing

Feces (>250 mg) was collected using a sterilized sample box and stored at −80°C within 2 h. Afterward, the collected samples were dissolved in peptone buffer to obtain 20% bacterial solutions. Tween 20 (0.5%) was added to the bacterial solutions, which were then centrifuged at 1,000 × *g* for 5 min at room temperature to collect supernatants and then for an additional 5 min at 10,000 × *g* to collect precipitates. The latter were washed with peptone buffer, dissolved in 50% glycerol, and stored at −80°C for later use.

Afterward, all the bacterial samples were centrifuged (9,000 rpm, 4°C) for 8 min before addition of 0.3 g (0.15 mm) and 0.1 g (0.7 mm) of zirconia/silica beads (Biospec Products, Bartlesville, OK, USA) and 1 mL of lysis buffer. The Multifunctional Tissue Homogenizer (6,300 × *g*, 35 s of bead beating, 45 s on ice; 6,300 × *g*, 35 s of bead beating, 45 s on ice; Bertin Technologies, Aix-en-Provence, France) was used to homogenize samples, which were then centrifuged for 5 min (15,000 × *g* at 4°C). Then, the supernatants were transferred to 2-mL Eppendorf (Hamburg, Germany) tubes, whereas 300 μL of lysis buffer was added to the precipitates. Then, the steps mentioned above were repeated to enable combination of the supernatants into 2-mL Eppendorf tubes. Ammonium acetate (300 μL) was mixed with the supernatants, which were centrifuged for 10 min (15,000 × *g* at 4°C). DNA was precipitated by addition of 1 volume of isopropanol, followed by centrifugation for 15 min (15,000 × *g* at 4°C). Afterward, the precipitates were washed with 1 mL of 75% EtOH, centrifuged (5 min, 15,000 × *g*, 4°C), dried (15 min, no heat, ‘Auto Run’ setting), and then dissolved in 100 μL of Tris-EDTA (TE) buffer (pH 7.5, 0°C, 30 min). The DNA solutions were reacted with RNase A (10 mg/mL; Sangon Biotech, Shanghai, China) before purification using an Ezup^®^ Column Bacterial Genomic DNA Extraction Kit (Sangon Biotech) and elution in CE buffer (a buffer for DNA dissolution, 50 μL), followed by storage at −20°C.

Frozen samples were sequenced to amplify the V4 region of 16S ribosomal RNA. Sequencing was achieved using a standard protocol for real-time reverse transcription-quantitative polymerase chain reaction employing a universal bacterial forward primer 515F (5′-GTGCCAG CMGCCGCGGTAAN-3′) and reverse primer 806R (5′-GGACTACHVGGGTW TCTAAT-3′). Samples in which amplification was successful were provided by BGI (Beijing, China) as the positive control and sterile deionized water served as the negative control. Raw data were filtered to delete low-quality sequences, and then, paired-end reads were added to tags by the Fast Length Adjustment of Short reads program (FLASH, v1.2.11) to get the tags (11). The tags were clustered into OTUs with a cutoff value of 97% using UPARSE software (v7.0.1090) (12), and chimera sequences were compared with the Gold database using UCHIME (v4.2.40) (13) to detect. Then, OTU representative sequences were taxonomically classified using Ribosomal Database Project (RDP) Classifier v.2.2 with a minimum confidence threshold of 0.6 and trained on the Greengenes database v201305 by QIIME v1.8.0 (14). The USEARCH global (15) was used to compare all Tags back to OTU to get the OTU abundance statistics table of each sample. The number of operational taxonomic units (OTUs) can be used to show the abundance of a particular species (16).

Phylogenetic Investigation of Communities by Reconstruction of Unobserved States (PICRUSt; http://picrust.github.io/picrust) was used to obtain three levels in the metabolic pathway and to ascertain the abundance table of each level (17). PICRUSt stores the orthology information from the Kyoto Encyclopedia of Genes and Genomes (KEGG) database corresponding to the Greengene ID, which can be used to obtain the Kegg Orthology (KO) corresponding to the OTUs for each sample (18). The data-library information from the KEGG database and OTU abundance were used to calculate the abundance of each functional category.

### Statistical analyses

Differences in bacterial abundance between groups were observed through partial least-squares discrimination analysis (PLS-DA) based on the OTU. The relative abundance of OTUs was analyzed using the linear discriminant analysis effect size (LEfSE) algorithm to identify taxa which could display significant differences in the two groups. The α-diversity of GM from each pregnant woman was assessed by the Simpson Index, Shannon Index, Sobs Index, and Chao Index. Significant differences between the two groups were analyzed by the Mann–Whitney *U*-test for non-parametric data. The relative abundance of GM was calculated at phylum, family, and genus levels between the two groups. HemI 1.0 was used to create heatmaps. SPSS 22.0 (IBM, Armonk, NY, USA) was employed for all statistical analyses. *P* < 0.05 was considered significant.

## Results

### Patients

The diagnosis of PE was based on the diagnostic criteria set by the American Congress of Obstetricians and Gynecologists (2). The clinical characteristics of all recruited pregnant women are shown in Table 1. Twelve women diagnosed as having PE during the third trimester were enrolled in the PE group. Eight normotensive healthy pregnant women were in the control group. There were significant differences in the antepartum body mass index (BMI), SBP, and DBP between the PE group and control group (*P* < 0.05) (Table 1), whereby the PE group had higher antepartum weight and blood pressure. There were no significant differences in age, gestational age, or progestational BMI between the two groups (*P* > 0.05).

**Table 1:** Clinical information of subjects.

	PE group (*n* = 12)	Control group (*n* = 8)	*P*
Age (years)	29.0±4.2	25.6±4.7	0.111
Gestational weeks	35.2±2.0	34.8±4.6	0.831
Pregestational body mass index (BMI) (kg/m^2^)	25.6±5.9	19.8±3.0	0.0503
Antepartum BMI (kg/m^2^)	30.4±4.9	24.6±3.3	0.005*
Systolic blood pressure (mmHg)	152.8±13.4	121.8±8.6	<0.001**
Diastolic blood pressure (mmHg)	100.5±10.0	72.3±6.0	<0.001**

Data are expressed as the mean ± SD. The statistically significant difference between the PE group and control group are *P* < 0.05 (*P < 0.05; **P < 0.01).

### Bacterial diversity in GM

To analyze the differences of GM between the two groups, 961,060 tags from 20 stool samples were obtained (average of 48,053±1,558 tags per sample). All tags were clustered into 1,227 OTUs.

**Fig. 1:**
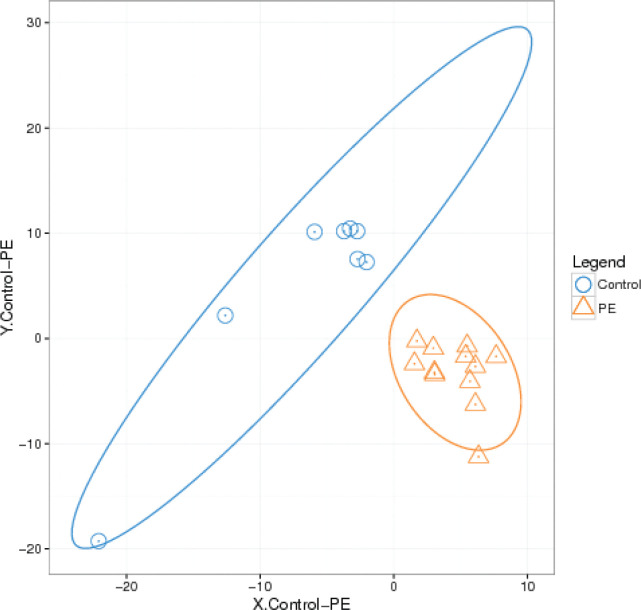
The β-diversity of the microbial communities in the two groups.

The results of *β*-diversity based on PLS-DA are shown in Fig. 1. Differences in GM communities between the two groups were documented. The distribution of samples from the PE group was more concentrated, whereas the distance between samples in the control group was longer. Hence, differences in the individual composition of GM were larger in the control group, and the similarities were smaller.

**Fig. 2:**
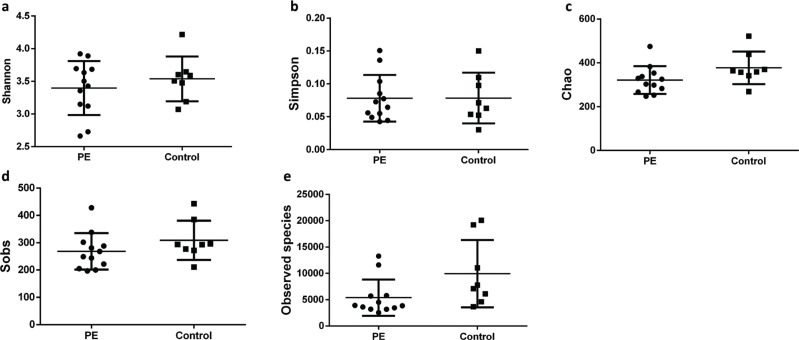
The altered biodiversity of gut microbiota in the PE group compared with control group. The groups of PE (*n* = 12) and control (*n* = 8) were described in the Methods (a, b). The ecological diversity of gut microbiota in the PE and control groups was measured by Shannon Index and Simpson Index (c, d). The alpha-diversity, richness of gut microbiota, was determined by Chao Index, Sobs Index, and Observed Species Index.

We investigated the α-diversity of GM in the two groups as described by Su and colleagues (19), which represented the mean species diversity in the overall gut. We found no significant differences in GM of the two groups. In general, the Shannon Index is used to quantify the uncertainty, whereas the Simpson Index is used to measure the degree of concentration, when individuals are classified into different types (20). However, there were no significant differences between the PE group and control group in terms of these two indices (*P* > 0.05) (Fig. 2a, b), although the Shannon Index decreased in the PE group. The Chao Index and Sobs Index also showed no significant differences (*P* > 0.05) (Fig. 2c, d), but the Chao Index and Sobs Index decreased moderately in the PE group. Meanwhile, the Observed Species Index of the control group was moderately higher than that of the PE group, although the difference was not significant (*P* > 0.05) (Fig. 2e). Taken together, these results showed that the species diversity in the PE group decreased, but there was higher distribution uniformity of bacteria in the PE group.

### Specific differences in GM

We wished to further identify the differences in GM between the two groups. Hence, we analyzed the number and relative abundance of all microbiota at phylum, family, and genus levels from all samples.

**Fig. 3:**
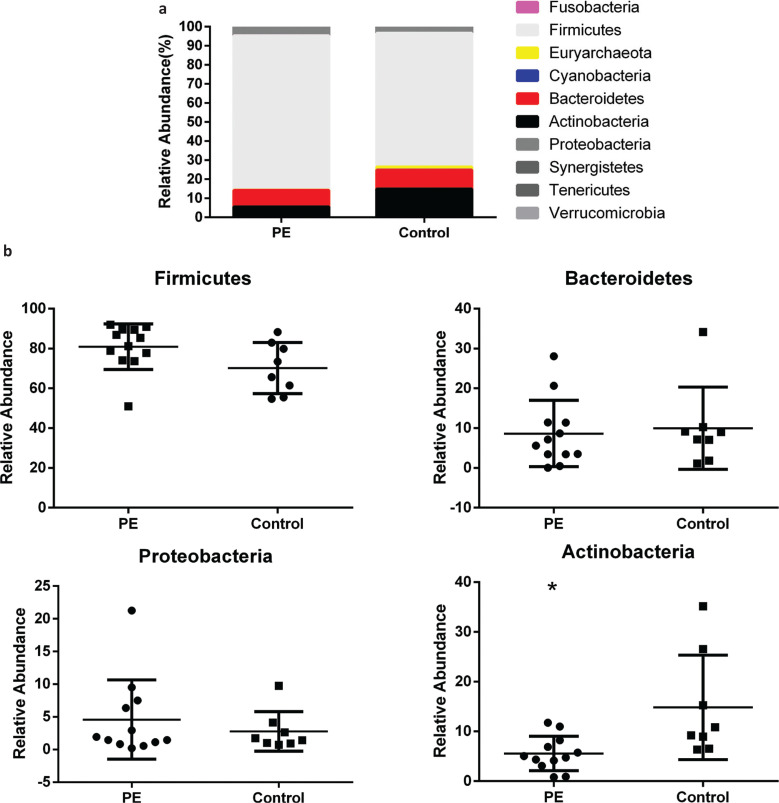
The relative abundance of gut microbiota at the phylum level in the two groups. a. The relative abundance percentage of gut microbiota at the phylum level. b. The relative abundance comparisons of four most abundant gut microbes between the two groups (*P = 0.042).

At the phylum level, the most abundant GM in the two groups were bacteria of the phylum Firmicutes (70.22–80.91%), Bacteroidetes (8.66–9.97%), Actinobacteria (5.56–14.86%), and Proteobacteria (2.8–4.6%) (Fig. 3a, Supplementary Table 1). The relative abundance of bacteria of the phylum Firmicutes in the PE group increased slightly compared with that in the control group, although the difference was not significant (*P* = 0.068) (Fig. 3b). The relative abundance of bacteria of the phylum Bacteroidetes was not significantly different between the two groups, although there was a decreasing trend in the PE group (*P* = 0.73) (Fig. 3b). In addition, the abundance of bacteria of the phylum Proteobacteria showed a slight increase but the difference was not significant (*P* = 0.77) (Fig. 3b). However, the relative abundance of bacteria of the phylum Actinobacteria was decreased significantly in comparison with that of the control group (*P* = 0.042) (Fig. 3b).

**Fig. 4:**
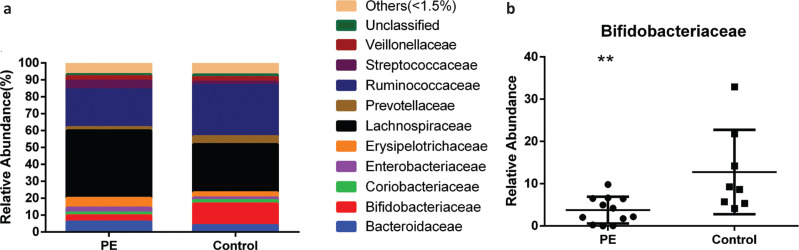
The relative abundance of gut microbiota at the family level of two groups. a. The relative abundance percentage of gut microbiota at the family level. b. The relative abundance comparisons of *Bifidobacteriaceae* between the two groups. (* *P* = 0.039).

The main families of bacteria in the stool samples were Lachnospiraceae, Erysipelotrichaceae, Bifidobacteriaceae, Ruminococcaceae, Bacteroidaceae, Streptococcaceae, Enterobacteriaceae, Veillonellaceae, Prevotellaceae, and Coriobacteriaceae (Fig. 4a, Supplementary Table 2). The relative abundance of bacteria of the family Bifidobacteriaceae was 3.75% in the PE group and 12.76% in the control group, respectively, and this reduction was significant in the PE group (*P* = 0.039) (Fig. 4b). The relative abundance of bacteria of the families Ruminococcaceae, Prevotellaceae, and Coriobacteriaceae in the PE group decreased compared with that in the control group, but the difference was not significant (*P* > 0.05). The relative abundance of bacteria of the families Lachnospiraceae, Bacteroidaceae, Erysipelotrichaceae, Streptococcaceae, Enterobacteriaceae, and Veillonellaceae in the PE group increased slightly compared with that in the control group, but the difference between the two groups was not significant (*P* > 0.05).

**Table 2:** Dietary situation of the two groups.

Group	Energy (kcal)	Protein (g)	Fat (g)	Carbohydrate (g)	Dietary fiber (g)
PE	2442.75 ± 483.59	95.99 ± 32.66	89.72 ± 32.67	319.05 ± 105.66	16.07 ± 5.84
Control	2265.23 ± 510.01	80.90 ± 28.70	87.88 ± 37.72	294.52 ± 72.33	17.10 ± 6.54
DRIs	2,250	85	50~75	281.25~365.63	25~30
*P*	0.524	0.386	0.924	0.635	0.637

DRIs, Dietary Reference Intakes (for pregnant women in third trimester). Data are expressed as the mean ± SD. The statistically significant difference between the PE group and control group are *P* < 0.05.

**Fig. 5:**
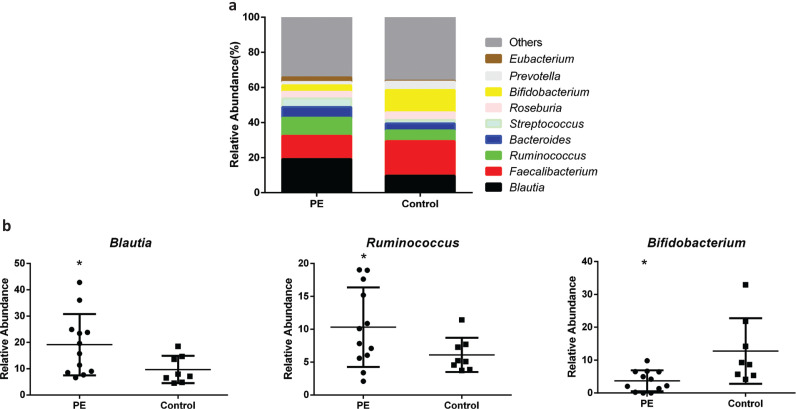
The β-diversity of the microbial communities in the two groups. a. The relative abundance percentage of gut microbiota at the genus level. b. The relative abundance comparisons of three gut microbes between the two groups (* *P*<0.05).

The major bacterial genera in GM were *Blautia*, *Faecalibacterium*, *Ruminococcus*, *Bacteroides*, *Streptococcus*, *Roseburia*, *Bifidobacterium*, *Prevotella,* and *Eubacterium* (Fig. 5a, Supplementary Table 3). Importantly, the abundance of bacteria of the *Blautia* was increased significantly in the PE group in comparison with that in the control group, with a relative abundance of 19.13% and 9.71%, respectively (*P* = 0.026) (Fig. 5b). Moreover, the relative abundance of bacteria of the genus *Ruminococcus* was increased significantly in the PE group compared with that in the control group (10.32% and 6.11%, respectively) (*P* = 0.048) (Fig. 5b). However, the relative abundance of bacteria of the genus *Bifidobacterium* demonstrated a marked reduction in the PE group in comparison with that in the control group (*P* = 0.038) (Fig. 5b). A similar downward trend was observed for bacteria of the genera *Faecalibacterium*, *Roseburia,* and *Prevotella* in the PE group compared with that in the control group (*P* > 0.05 for all). The abundance of the bacteria of the genera *Bacteroides*, *Streptococcus*, and *Eubacterium* was also increased, but the difference between the two groups was not significant (*P* > 0.05).

### Dietary analyses in pregnant women

We wished to explore the relationship between GM and the diet, so we analyzed the dietary situation in the two groups. The intake of energy, fat, and carbohydrate was all higher than the dietary reference intake (DRIs) for pregnant women in the third trimester in the two groups, whereas the intake of dietary fiber was lower (Table 2). The protein intake in the PE group was higher than the DRIs but, in the control group, the average protein intake was lower than the DRIs. Although there was no significant difference between the two groups (*P* > 0.05) in terms of the diet, an excess intake of macronutrients in the PE group was more obvious than that in the control group, but the intake of dietary fiber was lower.

### Correlation between clinical parameters and GM in pregnant women

**Fig. 6:**
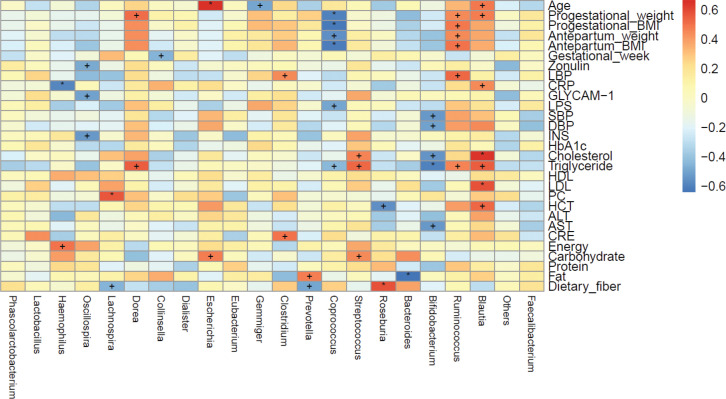
The correlations between different maternal clinical parameters and gut microbiota genus in the two groups. Spearman’s rank correlation coefficients and *P*-values for the correlations are shown (‘+’: *P*<0.05; ‘*’: *P*<0.01). LBP: lipopolysaccharide-binding protein; CRP: C-reactive protein; GlyCAM-1: glycosylation cellular adhesion molecules; LPS: lipopolysaccharide; SBP: systolic blood pressure; DBP: diastolic blood pressure; INS: insulin; HbA1C: glycosylated haemoglobin; HDL: high-density lipoprotein; LDL: low-density lipoprotein; PC: platelet count; HCT: hematokrit; ALT: alanine transaminase; AST: aspartate transaminase; CRE: creatinine.

We also investigated the correlation between maternal clinical parameters and the abundance of bacteria of certain genera in GM (Fig. 6). Remarkably, there was a significant positive correlation between maternal age and the relative abundance of bacteria of the genus *Blautia*, as well as the pregestational weight, hematocrit as well as levels of C-reactive protein and triglyceride (*P* < 0.05 for all). The level of low-density lipoprotein-cholesterol was highly significantly positively correlated with the relative abundance of bacteria of the genus *Blautia* (*P* < 0.01). The pregestational weight, pregestational BMI, antepartum weight, antepartum BMI, and levels of lipopolysaccharide-binding protein and triglyceride were significantly positively correlated with the relative abundance of bacteria of the genus *Ruminococcus* (*P* < 0.05 for all). However, the relative abundance of bacteria of the genus *Bifidobacterium* was significantly negatively correlated with the SBP, DBP, and levels of cholesterol and aspartate aminotransferase (*P* < 0.05 for all). The relative abundance of bacteria of the genus *Bifidobacterium* had a highly significantly negative correlation with the triglyceride level (*P* < 0.01).

### Prediction of function of GM using the KEGG database

**Fig. 7:**
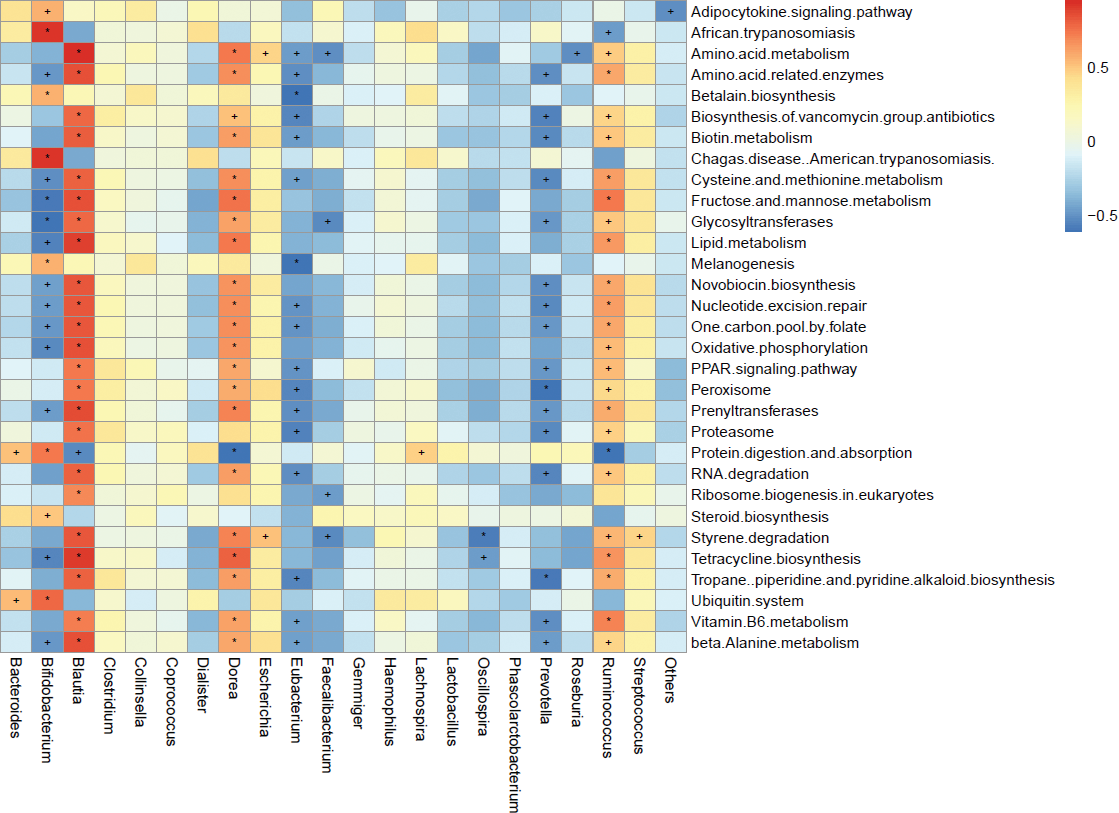
The correlations between different functional modules and gut microbiota genus. Spearman’s rank correlation coefficients and *P*-values for the correlations are shown (‘+’: *P* < 0.05; ‘*’: *P* < 0.01).

The functional prediction of GM using the KEGG database in the two groups was analyzed by PICRUSt (17) according to OTU profiles, and 270 functional modules from the KEGG database were obtained. Thirty-one functional modules (11.5%) were significantly different between the PE group and control group (*P* < 0.05) (Supplementary Table 5): 23 functional modules were reduced and eight functional modules were enriched in PE patients. The reduced functional modules included ‘PPAR signaling pathway’, ‘protein metabolism’, ‘RNA degradation’, ‘VB6 metabolism’, and ‘adipocytokine signaling pathway’. The enriched functional modules included ‘lipid metabolism’, ‘glycosyltransferases’, ‘biotin metabolism’, and ‘oxidative phosphorylation’. The functionally enriched genera *Blautia* and *Ruminococcus* in the PE group were significantly positively associated with all other enriched functional modules in the PE group, which showed their dominating functions in GM in the PE group (Fig. 7). The functionally reduced genus *Bifidobacterium* in the PE group was significantly negatively correlated with glucose metabolism, lipid metabolism, and oxidative phosphorylation, but significantly positively related with the digestion and absorption of protein.

## Discussion

We sequenced 16S rRNA of stool samples from 20 pregnant women. According to the testing of species diversity and OTUs, we demonstrated that there were differences in GM between healthy pregnant women and women with PE. The Observed Species Index was lower in the PE group than that of the control group (Figs. 1, 2), which suggested that there were some dominant bacteria and a lower uniformity of bacteria distribution in the PE group compared with that in the control group. Moreover, at phylum, family, and genus levels, the amount and relative abundance of GM from the PE group displayed substantial differences to those from the control group.

Endothelial dysfunction and subsequent activation of the coagulation cascade are very important in PE pathogenesis (2). There is growing evidence suggesting that an increased inflammatory response and an imbalance of immune regulation have vital roles in early-onset PE and severe PE (2, 3). Placenta dysfunction is also an important pathologic basis for PE (2). Amarasekara and colleagues demonstrated bacteria in the placental tissues of women with PE and supported the notion that these bacteria were one of the causes of PE (21).

Liu and colleagues revealed that, in women with PE, bacteria of the phylum Cyanobacteria had an increased abundance (1.07%) (8). We found a small decrease in the abundance of bacteria of the phylum Cyanobacteria in the PE group compared with that in the control group. However, we found that the control group had an increased abundance of bacteria of the phylum Tenericutes, a result that is consistent with that of the study from Liu and colleagues. Otherwise, the abundance of bacteria of the phylum Verrucomicrobia increased in the control group (8). Importantly, in our study, the relative abundance of bacteria of the phylum Actinobacteria was decreased significantly in the PE group. The abundance of bacteria from the family Bifidobacteriaceae and genus *Bifidobacterium* decreased significantly (*P* < 0.05) in the PE group. In general, the differences between our study and the study by Liu and colleagues may have been due to the influence of different geographic locations and dietary habits on GM. Nevertheless, the dominant different GM in women with PE reported by Liu and colleagues were similar with those reported by us.

Over the last decade, an increasing number of investigations have focused on the potential mechanisms by which intestinal microflora participate in the development of obesity (22), type-2 diabetes mellitus (23), hypertension (24), autism (25), and Parkinson’s disease (26). During pregnancy, intestinal microorganisms interact with the immune system and affect the maturation of the nervous system and hormone regulation (27). In GM, a balance in the abundance of bacteria from four phyla (Bacteroidetes, Firmicutes, Actinomycetes, and Proteobacteria) is crucial for the maintenance of intestinal immune homeostasis (28). In the intestines of healthy people, bacteria of the phyla Firmicutes and Bacteroidetes account for >90% of flora composition, and the ratio of their abundance plays a critical part in health (29). We found that bacteria of the phylum Firmicutes were the most prominent in GM, with >50% of the total abundance in the PE group and control group. Compared with that in the control group, the relative abundance of bacteria of the phylum Firmicutes in the PE group was increased slightly, whereas the abundance of bacteria of the phylum Bacteroidetes was essentially unchanged in the two groups. Therefore, the ratio of Firmicutes: Bacteroidetes was increased; alteration of this ratio may be one of the causes of PE. Moreover, the Firmicutes: Bacteroidetes ratio has been shown to be increased in a rat model of spontaneous hypertension and in people with systolic hypertension, data that are consistent with our findings in PE patients (29). In addition, the relative abundance of bacteria of the phylum Actinobacteria was decreased significantly in the PE group compared with that in the control group, whereas the abundance of bacteria of the family Bifidobacteriaceae and genus *Bifidobacterium* was also decreased significantly.

Studies have revealed that GM dysbiosis can contribute to hypertension and its complications (29), possibly by upregulating the inflammatory response. Levels of short-chain fatty acid (SCFAs) such as acetic acid, propionic acid, and butyric acid produced by GM are correlated with hypertension and influence vascular tone (30). GM of hypertensive rats have been shown to have fewer SCFA-producing bacteria compared with those of normotensive rats (30) and that a supply of probiotics reduces blood pressure (31). Hence, GM have important roles in the regulation of blood pressure. SCFA-producing bacteria can influence blood pressure by eliciting direct effects on vasodilation or through plasminogen activator inhibitor-1 (29). Bacteria of the genus *Bifidobacterium* are used commonly in probiotics and produce high levels of SCFAs, which can inhibit pathogen accumulation by reducing intestinal pH (32, 33). Similarly, our results showed that decreased abundance of bacteria of the genus *Bifidobacterium* was significantly negatively related to SBP, DBP, hyperlipidemia, and the level of aspartate aminotransferase, which may be causes of PE. We also showed that the abundance of bacteria of the genus *Blautia* was increased significantly (almost twofold) in the PE group compared with that in the control group and that its abundance was significantly positively correlated with age, pregestational weight, and hyperlipidemia. These results are similar with those of Lv and colleagues, who reported the abundance of bacteria of the genus *Blautia* to be enriched significantly in women with PE (34). Pregnancy at an older age, pregestational obesity, and abnormal lipid metabolism are risk factors for PE, having bacteria of the genus *Blautia* is positively correlated with a higher BMI (35) and consuming a high-fat diet can result in hyperlipidemia (36). Moreover, having bacteria of the genus *Blautia* is positively correlated with glucose intolerance (37) and is enriched in pregnant women who were overweight, obese, or who have excessive gestational weight gain (38). Crusell and colleagues found that gestational diabetes mellitus was related to an increased abundance of bacteria of the genus *Blautia*, which showed that enriched abundance of bacteria of the genus *Blautia* is associated with an unfavorable metabolic profile (39). The abundance of bacteria of the genus *Ruminococcus* has been reported to be enriched in women with gestational diabetes mellitus (39) or type-2 diabetes mellitus (40). It has been demonstrated that higher abundance of bacteria of the family Ruminococcaceae (which includes the genus *Ruminococcus*) is positively correlated with adverse metabolic situations (41) and leptin level (an adipocyte-derived hormone that is higher in PE patients) (41–43). In general, we found that decreased abundance of bacteria of the genus *Bifidobacterium* was negatively correlated with SBP and DBP and involved glucose metabolism, lipid metabolism, and the oxidative-phosphorylation pathway. However, an increased abundance of bacteria of the genera *Blautia* and *Ruminococcus* was positively correlated with obesity and dyslipidemia, which involved lipid metabolism, glycosyltransferases, biotin metabolism, and the oxidative-phosphorylation pathway. Therefore, we hypothesized that the alterations mentioned above in the abundance of bacteria of the genera *Bifidobacterium*, *Blautia*, and *Ruminococcus* might correlate with PE progression.

It is not uncommon for pregnant women to increase food consumption to meet the growth needs of the fetus, who are thereby prone to overnutrition. According to our dietary survey, PE patients had a higher intake of carbohydrate and fat, along with lower consumption of dietary fiber, compared with those of the control group. These actions were likely to cause disorders in glucose metabolism and lipid metabolism. Lower intake of dietary fiber would supply fewer prebiotics that are beneficial for probiotics, such as bacteria of the genus *Bifidobacterium.* Therefore, it may be useful to increase the intake of dietary fiber up to the DRIs (but not in excess) or take more probiotics, such as functional oligosaccharides (44, 45). Supplementation with bacteria of the genus *Bifidobacterium* may also be helpful for preventing PE. Our findings support the opinions of other scholars (46–49), but clinical trials are needed to confirm our findings.

## Conclusions

We reported, for the first time, GM changes in women with PE from East China. Our results indicated the potential for GM dysbiosis in PE patients and that they could be prone to suffer from metabolic syndrome. We speculate that alterations in the abundance of bacteria of certain genera (e.g. increased abundance of *Blautia* and *Ruminococcus* and decreased abundance of *Bifidobacterium*) were associated with PE development to some degree. Therefore, our data could help to monitor the health of pregnant women and may be helpful for preventing and assisting treatment of PE.
